# Equilibrium, Kinetic, and Diffusion Mechanism of lead(II) and cadmium(II) Adsorption onto Commercial Activated Carbons

**DOI:** 10.3390/molecules29112418

**Published:** 2024-05-21

**Authors:** Joanna Lach, Ewa Okoniewska

**Affiliations:** Faculty of Infrastructure and Environment, Czestochowa University of Technology, Brzeznicka 60a, 42-200 Czestochowa, Poland; ewa.okoniewska@pcz.pl

**Keywords:** activated carbon, adsorption, lead, cadmium

## Abstract

The adsorption of Pb(II) and Cd(II) on three commercial microporous activated carbons was analysed. Adsorption kinetics and statistics were investigated, and the results were described with different models. The highest values of the correlation coefficient R^2^ were obtained for the pseudo-second-order kinetics model for all ions tested and all sorbents used. The adsorption process was found to be determined by both diffusion in the liquid layer and intraparticle diffusion. The adsorption equilibrium is very well described by Langmuir, Temkin, Thoth or Jovanovic isotherm models. Based on the values of n from the Freundlich isotherm and K_L_ from the Langmuir isotherm, the adsorption of cadmium and lead ions was found to be favourable. The highest monolayer capacities were obtained during the adsorption of lead ions (162.19 mg/g) and for cadmium (126.34 mg/g) for activated carbon WG-12. This carbon is characterised by the highest amount of acid functional groups and the largest specific surface area. The adsorption efficiency of the tested ions from natural water is lower than that from a model solution made from deionised water. The lowest efficiencies are obtained when the process occurs from highly mineralised water.

## 1. Introduction

The presence of heavy metals in the environment has been discussed for many years. Heavy metal ions, such as Cd(II) and Pb(II), are among the toxic inorganic pollutants that can cause environmental problems even when present in low concentrations in surface or groundwater [1,2,3]. Their concentrations often exceed WHO recommendations in developing countries, even in drinking water [4]. Wastewater is the main, but not the only, source of these pollutants [5].

Cadmium is used in the steel industry, the plastics industry, the manufacturing of cement, batteries and pigments, and in electroplating [6]. It is harmful to humans and the environment, even at low concentrations, due to its accumulation in biological organisms. Excessive cadmium intake leads to kidney damage, chronic pulmonary problems, proteinuria, skeletal deformities, cardiovascular disease, muscle spasms, and testicular atrophy [7,8].

The World Health Organisation (WHO) set the maximum concentration of Cd in drinking water at 0.003 mg/L [9]. The methods used to remove cadmium include ion exchange, electrocoagulation, flocculation, coagulation and filtration, membrane processes, adsorption, and biosorption [6,10].

Lead is not essential for, and is highly toxic to, living organisms and the environment [11]. Consumption of lead-contaminated water can cause iron deficiency, constipation, anaemia, brain damage, cancer, and even death [11,12]. It is a heavy metal that is the most frequently analyzed in drinking water after arsenic [13]. It can be found in wastewater from, among others, the electroplating industry, the paint industry, the glass industry, and battery manufacturing [6]. The maximum permissible limit of Pb(II) in drinking water by WHO is 0.05 mg/L [9]. Effective methods for lead removal include chemical precipitation, membrane filtration, reverse osmosis, ion exchange, biosorption, and adsorption [6,14].

Adsorption on activated carbon appears to be a suitable method for removing metals at low concentrations due to, among other reasons, there being no need to introduce additional chemicals into the water, the lack of sediment formation, the simplicity of the process, the wide range of carbon sorbents, as well as its relatively low cost and high efficiency, high mechanical strength, and good abrasion resistance [6]. Activated carbons are highly variable in terms of their porous structure, the size of the specific surface area, and the chemical nature of the surface [15,16]. Depending on the production method and their subsequent modification, they have different functional groups (carboxyl, carbonyl, phenol, lactone, and quinone), giving activated carbons specific properties responsible for adsorbing a very wide range of pollutants.

Both commercial sorbents and low-cost sorbents made from waste materials are proposed for the removal of heavy metals from water [16,17,18,19].

The study analysed activated carbons used in water treatment plants to remove organic compounds. The activated carbons were evaluated in terms of their applicability to the removal of heavy metal ions differing, among other things, in their electrical charge. An analysis of the possibility of extending the area of application of commercial activated carbons to heavy metal ions allows for a more economical adsorption process.

The aim of this study is to analyse the potential for the sorptive removal of Pb(II) and Cd(II) ions on commercial activated carbons, propose process mechanisms, and determine the kinetics and statics of adsorption using different mathematical models. Different models of adsorption kinetics and statics were examined, and their suitability for describing adsorption statics and kinetics processes was assessed. The usefulness of the different models was evaluated in comparison to the most commonly used: Freundlich isotherms, Langmuir, and PFO and PSO kinetics equations. The effect of the presence of competing ions on the adsorption efficiency of Pb(II) and Cd(II) cations was also analysed.

## 2. Results and Discussion

### 2.1. Kinetics of Pb(II) and Cd(II) Adsorption

#### 2.1.1. Kinetics of Pb(II) Adsorption

**Figure 1:**
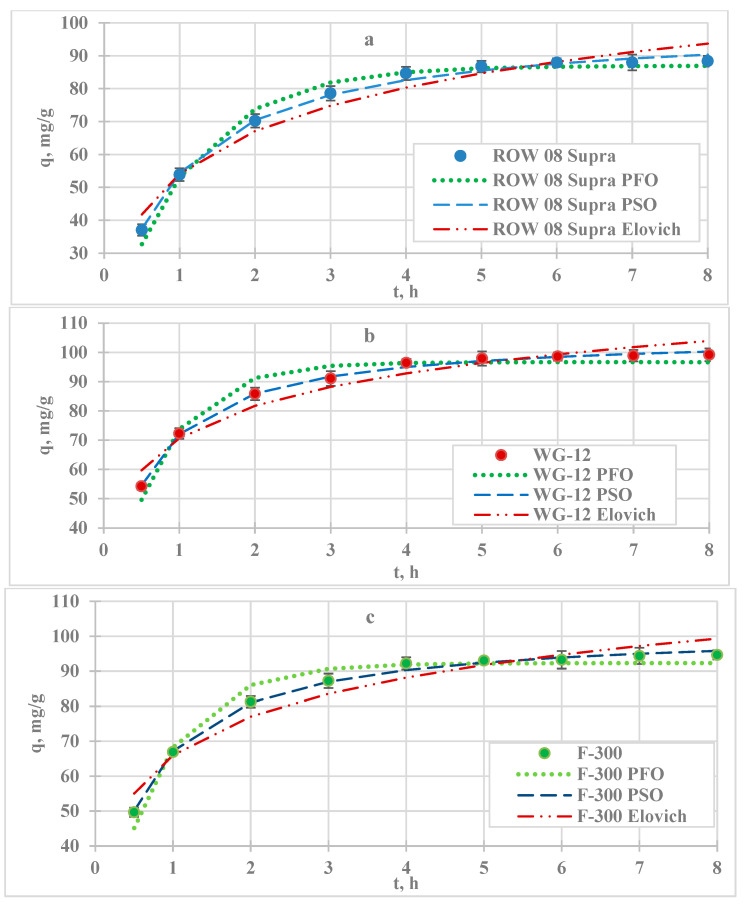
Kinetics of lead ion adsorption on activated carbons; C_0_ = 100 mg/L, pH = 6: (**a**) ROW 08 Supra; (**b**) WG-12; (**c**) F-300.

In the first stage of the lead adsorption study, the adsorption kinetics were analysed (Figure 1, Table 1). The removal of the Pb(II) cation was fastest in the first stage of the process. After 1 h, the adsorption efficiency of lead ions ranged from 39.7% *w*/*w* (F-300) to 57.8% *w*/*w* (WG-12). After 8 h, the adsorption efficiency ranged from 70.7% *w*/*w* (ROW 08 Supra) to 79.4% *w*/*w* (WG-12). The adsorption kinetics were fast in the first stage of the process, as readily available active sites were used.

The Pb(II) adsorption equilibrium establishment time. The study assumed that the adsorption equilibrium is established when the concentration of the tested metal changes by less than 1% *w*/*w* of the initial concentration (0.5 mg/L) in the subsequent measurement. Differences in final concentrations of less than 0.5 mg/L between the two tests were obtained after 5 h for the ROW 08 Supra and F-300 carbons and after 6 h for the WG-12 activated carbon. The adsorption equilibrium establishment time was longest on ROW 08, even though the adsorption rate calculated from the PSO kinetics equation (k_2_) for this sorbent was highest among the analysed activated carbons. However, this sorbent adsorbed the highest amounts of the study ion. Other researchers obtained very different times of adsorption equilibrium establishment depending on the sorbent used, e.g., 0.5 h [20], 4 h [21], and even 24 h [22].

The results were analysed using three models of adsorption kinetics (Table 1). Considering the correlation coefficients R^2^ and the errors ARE (average relative error), the models can be arranged as follows: PSO > PFO > Elovich. However, for all models, the correlation coefficient R^2^ is high (over 0.94). Researchers most often use the PFO and PSO models to describe the kinetics of lead ion adsorption (Table 1). In most cases, the PSO model describes the study results better than PSO, Elovich, and IPD. A similar trend was observed when analysing the adsorption in this study. Comparing the k_2_ adsorption rate coefficients, the fastest adsorption process occurred on ROW 08 Supra WG-12 and the slowest process on ROW 08 Supra. For the other two models, analogous results were obtained: the adsorption was fastest on WG-12 carbon (based on k_1_ and α coefficients) and slowest on ROW 08 Supra. The speed of adsorption influences the equilibrium establishment time, which is shortest for WG-12 activated carbon.

#### 2.1.2. Kinetics of Cd(II) Adsorption

The studies of adsorption kinetics and statics were carried out using solutions at pH = 6, at which cadmium adsorption is optimal [23].

**Figure 2:**
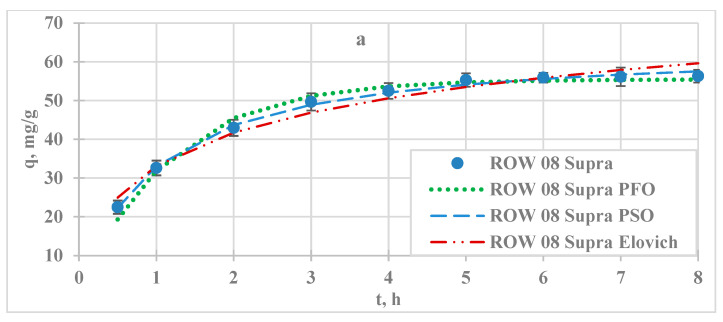

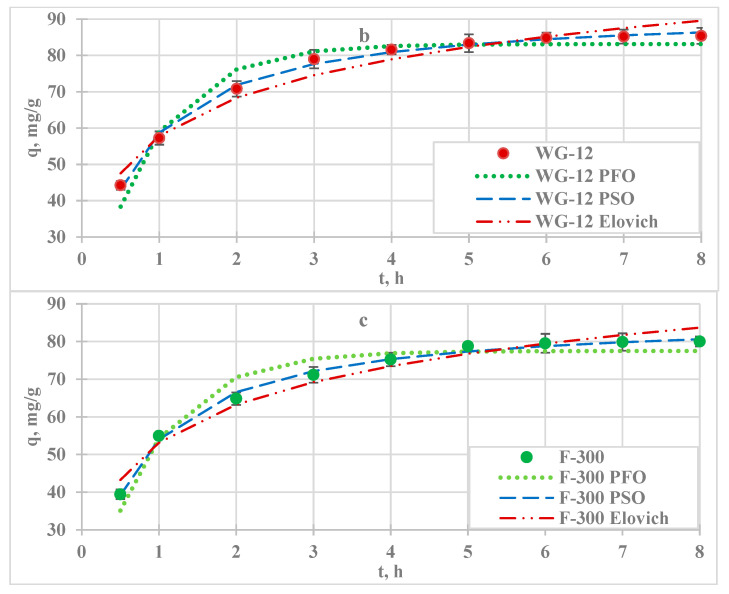
Kinetics of lead cadmium adsorption on activated carbons; C_0_ = 100 mg/L, pH = 6: (**a**) ROW 08 Supra; (**b**) WG-12; (**c**) F-300.

Based on adsorption kinetics studies, the adsorption equilibrium establishment time was found to be shortest for F-300 activated carbon (5 h). For WG-12 and ROW 8 Supra, it was 6 h (Figure 2). These times were similar to those obtained for lead sorption. The literature reports different times of adsorption equilibrium establishment, e.g., 20 min [24], 2 h [23], and 6 to 24 h [25].

The obtained adsorption kinetics results were described using the PFO, PSO, and Elovich models (Table 2). The best fit of the study results, assessed on the basis of R^2^ and E, was obtained for the PSO model (R^2^ ranging from 0.995 to 0.996 and ARE ranging from 1.11 to 1.37%). The PFO and PSO models were semi-empirical and it was difficult to suggest an adsorption mechanism on their basis. The PSO model suggests the importance of chemisorption. The adsorption rate determined by the k_1_ coefficient was similar on the studied microporous activated carbons; it was 0.016 h^−1^ for ROW 08 Supra and 0.019 h^−1^ for F-300 and WG-12. In the case of the other two models analysed, it was not possible to determine unequivocally which one described the study results better, as it depended on the sorbent used. However, they described the test results with a fairly high degree of accuracy, with R2 ranging from 0.942 to 0.981. Most of the studies reported in the literature use the PFO and PSO models, but the PSO model is usually the one that better describes the results of adsorption kinetics studies (Table 2).

### 2.2. Analysis of Mass Transfer in the Adsorption Process

The adsorption process consists of four mass transport phases associated with porous adsorbents. The first phase, called ‘bulk transport’, occurs immediately after the sorbent is transferred into solution. It is very fast (it does not significantly affect the adsorption time) and is therefore often neglected due to being seen as irrelevant in considerations [26]. The second phase, film diffusion, is much slower. In this phase, the adsorbate molecules are transported from the liquid phase to the surface of the activated carbon. This takes place in the hydrodynamic boundary layer or film. The third stage involves the diffusion of adsorbate molecules from outside the adsorbent into the pores of the adsorbent and/or along the surface of the pore walls (intraparticle diffusion). This stage occurs slowly. The last stage, the adsorption of the molecule, is very fast and can be skipped like stage one. The second stage and the third stage, as the slowest, are the stages that determine the adsorption time [27].

**Figure 3:**
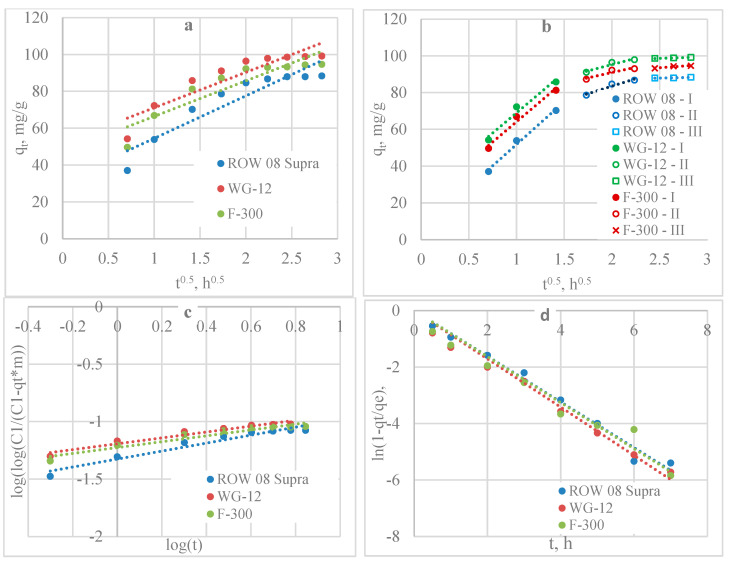
Mass transfer model analysis of Pb(II): (**a**) Weber-Morris; (**b**) Weber-Morris; (**c**) Bangham’s and Burt model; (**d**) liquid film diffusion model.

**Figure 4:**
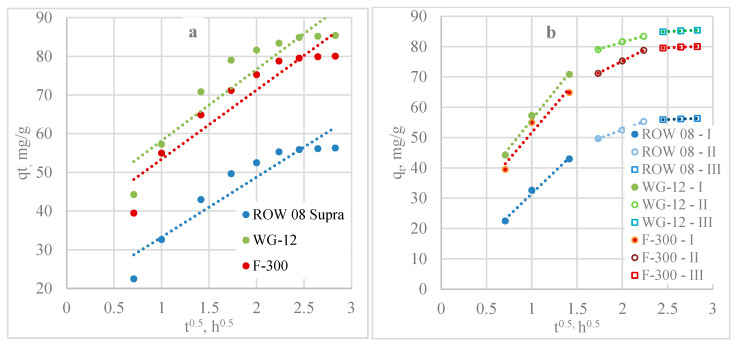

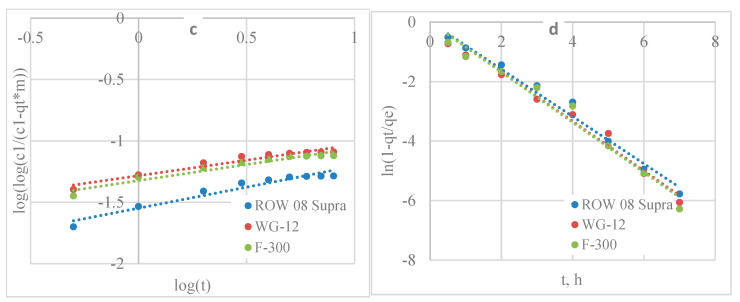
Mass transfer ^model^ analysis of Cd(II): (**a**) Weber-Morris; (**b**) Weber-Morris; (**c**) Bangham’s and Burt model; (**d**) liquid film diffusion model.

The Weber-Morris model assumes that the adsorption process is controlled by intraparticle diffusion if the graph of qt(t^1/2^) is a straight line and passes through the origin of the coordinate system. The graphs of the relationship qt(t^1/2^) for both Pb(II) (Figure 3a) and Cd(II) ions (Figure 4a) do not pass through the origin of the coordinate system and are characterised by low values of R^2^ (R^2^ from 0.8297 to 0.8657 for Pb(II) and from 0.8624 to 0.8876 for Cd(II)) (Table 3 and Table 4). This indicates that intraparticle diffusion is not the only phase limiting the adsorption rate.

Another model used to determine whether pore diffusion is the mechanism controlling the rate of adsorption is the Bangham and Burt model. A line plot of this model for Pb(II) adsorption is shown in Figure 3c, and the K_b_ and α constants are shown in Table 3. For Cd(II), the results are shown in Figure 4c and in Table 4. For this model, higher R^2^ coefficients were obtained for both heavy metals (from 0.9282 to 0.9464 for Pb(II) and from 0.9386 to 0.9432 for Cd(II)) compared to the Weber-Morris model. The results show that diffusion in the pores influenced the adsorption kinetics.

The Weber-Morris diffusion model is also presented, dividing the graphs into rectilinear sections. Three stages were separated for each activated carbon and both adsorbates (Figure 3b and Figure 4b, Table 3 and Table 4). The first stage characterises mass transfer as a result of Pb(II)/Cd(II) diffusion on the outer surface of the activated carbons. The second section characterises the mass transfer due to Pb(II)/Cd(II) diffusion inside the pores. The third section, almost parallel to the *x*-axis, shows the equilibrium state in which the Ac surface is fully saturated with Pb(II)/Cd(II) molecules. These results show that the mass transfer rate was influenced by both steps.

A liquid film diffusion model was also analysed (Figure 3d and Figure 4d, Table 3 and Table 4). This model with a high R^2^ (0.9497 to 0.9841 for Pb(II) and 0.9737 to 0.9840 for Cd(II)) describes the mass transfer of lead and cadmium ions on all activated carbons. These results confirm the significant influence of liquid film diffusion on adsorption kinetics.

The influence of liquid film diffusion and intraparticle diffusion has also been observed by other researchers analysing heavy metal adsorption [28,29,30].

### 2.3. Equilibrium of Pb(II) and Cd(II) Adsorption

The following models are used to describe the adsorption isotherms of the ions studied: two-parameter models Freundlich (F), Langmuir (L), Temkin (T), Jovanovic (J), and Halsey (H) and three-parameter models Redlich−Peterson (R-P) and Toth (To). Among the most commonly used are the Freundlich and Langmuir models. However, a better fit to the study results can often be obtained using other models. Each of the models used is characterised by different assumptions. All models used (except the Halsey model) assume monolayer sorption. Homogeneity of the surface is assumed by the Langmuir model. However, it assumes the possibility of interactions, resulting in a multilayer effect. A similar assumption is made for the Toth isotherm. The Freundlich and Temkin models assume adsorption on a heterogeneous surface. Other equations assume adsorption on both homogeneous and heterogeneous surfaces. Some of the isotherms assume physical adsorption—Jovanovic, Toth— or chemical adsorption—Temkin. Other models describe both physical and chemical adsorption (Freundlich, Langmuir, Redlich−Peterson). The analysis of isotherms can therefore suggest an adsorption mechanism. By determining the equations of the various adsorption models, further information can be obtained from the data obtained. From the Langmuir, Jovanovic and Toth models, the maximum capacity of activated carbons—q_m_—can be determined. From the Langmuir isotherm, for example, the coefficient RL can be determined, while, from the Freundlich isotherm, the coefficient n can be determined; additionally, whether the adsorption is favourable can then be determined from these aspects. Analysis of the coefficients of different isotherms can provide very different information for characterising adsorption [31,32].

#### 2.3.1. Equilibrium of Pb(II) Adsorption

**Figure 5:**
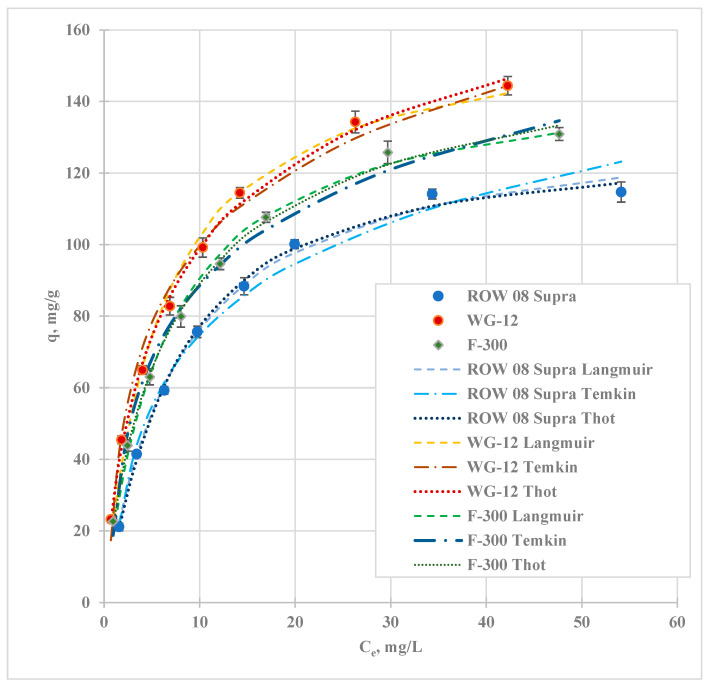
Isotherms of adsorption of Pb(II) ions.

Isotherms of lead adsorption in the study commercial activated carbons were analysed. The results are presented in Figure 5, and the calculated coefficients of the seven models are shown in Table 5. To assess the fit of the isotherm model to the test results obtained, the correlation coefficient R^2^ is most commonly used. Considering the coefficient R^2^, the models can be arranged as follows: T > To > L > J > F = R-P > H. The study also analysed the value of the following errors: ARE—average relative error; sum of squares of errors (SSE); Chi-squared statistics (λ^2^), sum of the absolute errors (SAE), and the hybrid fractional error function (HYBRID). When analysing the average relative error value, activated carbons can be ranked in the same way as when they were assessed based on their R^2^ value. For the analysis of SSE, λ^2^, SAE, and HYBRID errors, it was found that the lowest error values were obtained for the Toth isotherm. Slightly higher error values were obtained for the Temkin and Langmuir isotherms. The highest error values were obtained for the Halsey model, which assumes multilayer sorption. In Table 5, the highest R^2^ values and the lowest analysed error values are bolded. The comparison of these values was performed separately for each activated carbon. The three models that best describe the results obtained are additionally presented graphically in Figure 5. The correlation coefficients for these models on the activated carbons tested range from 0.990 to 0.999 and the error ARE from 0.33 to 6.81%. On the basis of the isotherms obtained, it is not possible to determine unequivocally the adsorption mechanisms, as the Temkin model assumes chemical adsorption, the Toth model assumes physical adsorption, and the Langmuir model applies to both chemical and physical adsorption. These models assume monolayer sorption. The Halsey model is the only one that assumes multilayer sorption and has the lowest correlation coefficients. The Temkin and Toth isotherms assume that adsorption occurs on a heterogeneous surface, while the Langmuir isotherm assumes that adsorption occurs on a homogeneous surface. In the literature, authors mostly use the Langmuir and Freundlich models to describe Pb(II) adsorption results. On the basis of the studies cited in Table 6, it is not possible to determine unequivocally which of the models describes the study results better. In some cases, R^2^ is higher for the Freundlich model while, in others, it is higher for the Langmuir model. Other models are used very rarely to describe the results of Pb(II) ion studies.

The Langmuir, Toth, and Jovanovic isotherm models can be used to determine the capacity of the monolayer. Based on the q_m_ values (the Langmuir model), the carbons can be arranged as follows: WG-12 > F-300 > ROW 08 Supra (q_m_ ranging from 135.94 to 162.19 mg/g). The q_m_ values from the Langmuir isotherm are similar for all carbons. The q_m_ values from the Toth model differ to a much greater extent (q_m_ ranging from 127.6 for ROW 08 Supra to 209.83 mg/g for WG-12). The qm values determined using the Jovanovic model are lower and range from 113.89 (for ROW 08 Supra) to 137.85 mg/g (for WG-12). Analysing the K_L_ values, it can be concluded that the highest affinity of lead ions to the surface was obtained for WG-12 activated carbon and the lowest for ROW 08 Supra. The calculated R_L_ values prove that the adsorption of lead ions is favourable on the commercial activated carbons used in the study (R_L_ = 0.9 ÷ 0.99).

Comparing the obtained adsorption capacities with the literature reports, it can be concluded that average Pb(II) sorption capacities were obtained using commercial activated carbons. The studies on the adsorption of Pb(II) were conducted using solutions at pH = 6, which was considered optimal for the adsorption of Pb^2+^ cations [33]. Under these conditions, the surface of the activated carbon was positively charged (pH_PZC_ ranging from 6.4 to 6.6). However, the charge was small because the pH of the solution was close to the pH_PZC_ of the studied sorbents.

The adsorption on the tested activated carbons can occur based on the following mechanisms [20,34]:

- on amphoteric and alkaline activated carbons:

AC: + Pb^2+^ → AC: Pb^2+^ (Cπ − cation interactions) (1)

- on “acidic” activated carbon

AC–COOH + Pb^2+^ + H_2_O → AC–O–COOPb^+^ + H_3_O^+^ (2)

(AC–COOH)_2_ + Pb^2+^ + 2H_2_O → (AC–COO)_2_Pb + 2H_3_O^+^ (3)

AC–OH + Pb^2+^ + H_2_O → AC–OPb^+^ + H_3_O^+^ (4)

In addition to ion exchange and complexation, lead can be removed on carbons by the deposition of oxides and hydroxides.

In connection with the presented mechanisms of lead ion adsorption, the number of functional groups was examined (carbon characterisation is included in the test methodology). The highest number of acid groups is on the surface of the activated carbon that has the best absorption properties (WG-12) and the smallest number is on the activated carbon that has the poorest absorption properties (ROW 08 Supra). Analysing the types of functional groups, it can be noticed that activated carbon WG-12 has the most carboxyl groups and ROW 08 Supra has the least. Carboxyl groups under the tested conditions (pH = 6) are dissociated and may participate in the removal of lead cations [26,35].

**Figure 6:**
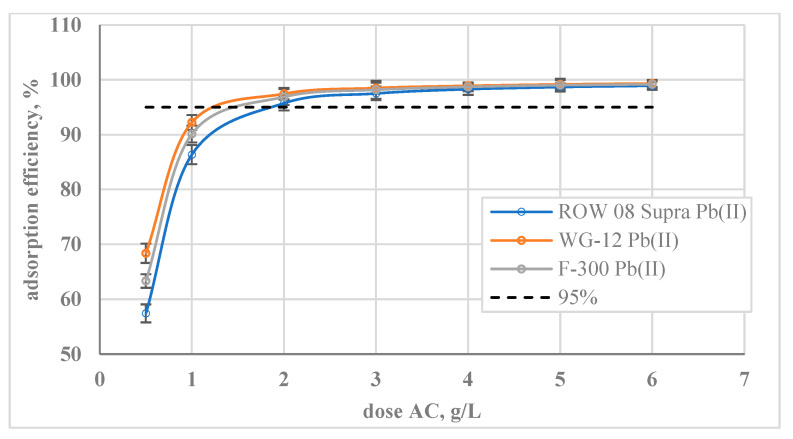
Effect of activated carbon dose on the adsorption of Pb(II) ions; C_0_ = 100 mg/L, pH = 6.

The effect of doses on the adsorption efficiency was also analysed (Figure 6). The lowest of the analysed doses (0.5 g/L) resulted in the removal of 57% *w*/*w* of Pb(II) ions for ROW 08 Supra activated, carbon, 63% *w*/*w* for F-300, and 68% *w*/*w* for WG-12. A dose of 2 g/L resulted in more than 95% *w*/*w* removal of the analysed ions on all activated carbons.

**Table 6:** Comparison of Pb(II) adsorption results based on the literature reports.

Activated Carbon	Max. q_m_, mg/g	Adsorption Isotherms Equations Tested	Equations of Adsorption Kinetics Studied	Ref.
From eucalyptus saw	128.21	L > T > F	PSO > PFO > Elovich	[36]
Polypyrrole-based AC	50	F > L	PSO > PFO	[21]
From black cumin seeds	15.7	F > L	PFO > IDP > PSO PSO > IDP > PFO PSO = IDP > PFO	[37]
From used tires	322.50	L > D-R > T > F	PSO > PFO	[22]
Commercial	42.5	F > L > T > D-R		
From rapeseed straw	253	F > L	PSO > PFO	[38]
Commercial	146			
From the Endocarp Waste of Gayo Coffee	434.78	L > D-R > F	PFO > PSO (2 studies) PSO > PFO (1 study)	[39]
From synthetic sewage with activated carbon	122.07	F > L	PSO > PFO	[40]
Of doum palm shell	500	L > F	PSO	[41]
From molasses	303	L > F	-	[42]
From lignocellulosic waste	232.56	L > T > F	PSO > IDP > PFO	[43]

#### 2.3.2. Equilibrium of Cd(II) Adsorption

**Figure 7:**
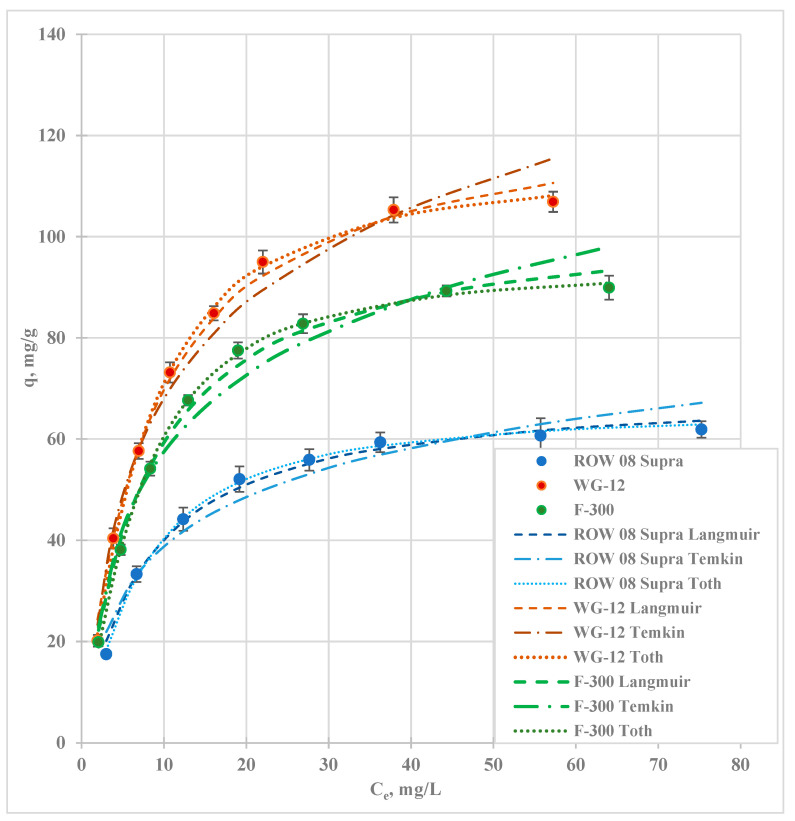
Isotherms of adsorption of Cd(II) ions.

Figure 7 shows the Cd(II) adsorption isotherms. Table 7 shows the calculated coefficients of the isotherm models. The figure shows the experimental points and plots of the calculated models, which have the highest values of the correlation coefficient R^2^ and the lowest errors ARE, SSE, λ^2^, SAE, and HYBRID, i.e., Toth, Langmuir, and Temkin (R^2^ ranging from 0.991 to 0.998). Cadmium adsorption is also well described by the Jovanovic isotherm. In most cases, lower error values are obtained for this isotherm compared to the Langmuir and Temkin isotherms. All these models assume monolayer adsorption. However, there are no other assumptions that are the same for all three isotherms. The Langmuir model assumes surface homogeneity, the Temkin model assumes heterogeneity, and the Toth model, which is based on Langmuir assumptions, applies to both homogeneous and heterogeneous surfaces. The Temkin model assumes chemical sorption, the Toth model assumes physical sorption, and the Langmuir model assumes both chemical and physical sorption. The good fit of these models, despite them being different, demonstrates the complexity of the processes responsible for the adsorption of cadmium on the carbon sorbents used in the study.

The analysis of the literature reports shows that Langmuir and Freundlich isotherms are most commonly used to describe cadmium adsorption. In most cases, the Langmuir equation describes the study results better than the Freundlich model (Table 8). In this study, a clearly better fit of the experimental results was obtained with the Langmuir model.

The q_m_ values calculated using the Langmuir isotherm range from 69.91 mg/g (ROW 08 Supra) to 126.34 mg/g (WG-12) (Table 7). The q_m_ values calculated on the basis of the Jovanovic isotherm are slightly lower, while those calculated on the basis of the Toth isotherm are higher. Based on the capacity of the monolayer, the activated carbons can be arranged in the following order: WG-12 > F-300 > ROW 08 Supra. The differences between these carbons are large. Similarly to the adsorption of lead ions, the arrangement determined by the Cd(II) sorption rate is not in line with the arrangement of the carbons by size of the specific surface area. The WG-12 activated carbon, which sorbs the most cadmium, has the highest number of acidic groups determined by the Boehm method, while ROW 08 Supra, which most poorly sorbs Cd(II) ions, has the smallest number of acidic groups. The K_L_ values for all sorbents are similar (K_L_ ranging from 0.123 to 0.135), indicating a similar affinity for cadmium in all the activated carbons used in the study. The analysis of the R_L_ values from the Langmuir isotherm and 1/n from the Freundlich model shows that the cadmium adsorption process is favourable on all the activated carbons used in the study. The ranges of parameter R_L_ values for all activated carbons fitted in a range from 0 to 1 (from 0.90 to 0.99), which means that the adsorption in the tested concentration range was favourable. The reciprocal of the coefficient n (1/n) indicates the degree of diversity of active areas on the surface of activated carbon. The obtained values of 1/n (from 0.357 to 0.362) are similar for the tested adsorbents.

The mechanisms responsible for the adsorption of cadmium on activated carbons are analogous to those for lead. They can take place according to Equations (1)–(4). The amount of carboxyl groups that are dissociated at pH = 6 affects the amount of cadmium adsorption. Ion exchange and complexation are the main mechanisms responsible for cadmium adsorption [40]. According to Al-Saadi et al. (2013), the adsorption of cadmium ions is mainly related to the presence of carboxyl groups on the caron surface; however, other acid functional groups are also involved in the process [44].

**Figure 8:**
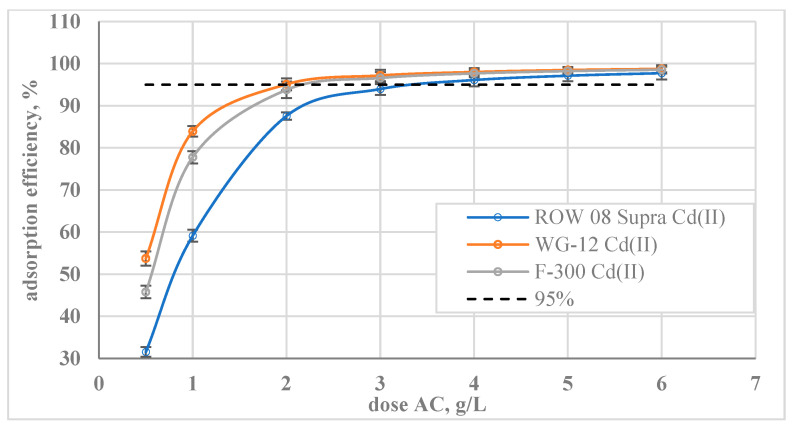
Effect of activated carbon dose on the adsorption of Cd(II) ions; C_0_ = 100 mg/L, pH = 6.

The effect of doses on the adsorption efficiency of cadmium ions was also assessed (Figure 8). The lowest of the analysed doses (0.5 g/L) resulted in the removal of 32% *w*/*w* of Cd(II) ions for ROW 08 Supra activated carbon, 46% *w*/*w* for F-300, and 54% *w*/*w* for WG-12. A dose of 2 g/L of WG-12 activated carbon resulted in more than 95% removal of cadmium ions. For the other activated carbons, the doses had to be higher, e.g., 3 g for F-300 and 4 g for ROW 08 Supra.

**Table 8:** Comparison of Cd(II) adsorption results based on the literature reports.

Activated Carbon	Max. q_m_, mg/g	Adsorption Isotherms Equations Tested	The Equations of Adsorption Kinetics Studied	Ref.
From synthesis wastewater with activated carbon	119.41	L > F	PSO > PFO	[40]
From coconut shell	135.76	L > F > T	PFO > PSO	[25]
Commercial	25.13	L > F	PSO > PFO	[24]
Silkworms’ feces-based	80	L > F	PSO > PFO > Weber-Moris	[45]
Commercial	682.5	F > L > T	PSO > PFO > IPD	[46]
From phragmites australis	62.11	L > F	-	[47]
Commercial	178.5	L > F	-	[48]
Of doum palm shell	125	L > F	PSO	[41]
From oil palm shell	227.27	L > T > F	PSO > Elovich > PFO	[49]
From shea nut	5.46	F > L	PSO > PFO	[50]
From activated carbon/zirconium oxide composite	200	L > F	PFO > PSO	[51]
Magnetite–diatomite nanocomposite	31.46	T > L = F > D-R	PSO > PFO > IPD	[52]

### 2.4. Adsorption of Tested Ions from Natural Waters

The effect of the composition of natural waters on the adsorption efficiency of Pb(II) and Cd(II) ions on WG-12 activated carbon was assessed (Table 9). Tests were carried out on waters whose chemical composition varied considerably. For the adsorption of Pb(II) and Cd(II) cations, competing cations Ca^2+^, Mg^2+^, Na^+^, K^+^ are present in natural waters. Their concentrations (especially of calcium cations) are many times higher than that of the removed heavy metal cations. It was found that the higher the sum of competing cations, the lower the adsorption efficiency of Pb^2+^ and Cd^2+^ ions. The reduction in adsorption efficiency is small if adsorption occurs from spring and low-mineralised waters (by less than 4% *w*/*w*), despite the fact that the sum of the cations in these waters is several times greater than the removed heavy metal cations. For medium- and high-mineralised waters, the sum of cations is 13 and 39 times greater than that of Pb(II) or Cd(II) ions. Adsorption efficiencies lead from such solutions are 2–25% *w*/*w* lower compared to adsorption from deionised water. Large differences in adsorption efficiency are also observed for cadmium (3 to 18%). Despite the high mineralisation of the water and the high amount of competing ions, even when adsorbed from highly mineralised water, the removal efficiency for Pb(II) and Cd(II) ions is higher than 60% *w*/*w*. SEM/EDX studies (Table 10) confirm greater adsorption of lead than cadmium.

### 2.5. Mechanism of Adsorption of Pb(II) and Cd(II) ions from Aqueous Solutions

Lead and cadmium occur in these solutions as divalent cations, Pb^2+^ and Cd^2+^. In both anion and cation adsorption, the chemical structure of the specific surface is an important factor.

Adsorption of lead and cadmium cations, among others, follows Equations (2) and (3). As the study is conducted from a solution with pH = 6, the surface of the activated carbons has a slightly positive charge (pH_PZC_ of 6.4 to 6.6) (Table 11). Under these conditions, the carboxyl groups (pKa of 3 to 6) and some of the lactone groups (pKa of 7 to 9) are in dissociated form. There is an electrostatic attraction between the lead and cadmium cations and the deprotonated carboxyl/lactone groups. Another mechanism is the possibility of ionic bonding with the C–O group. This adsorption mechanism, according to studies by, for example [26,43,44], is responsible for the adsorption of lead and cadmium ions.

**Figure 9:**
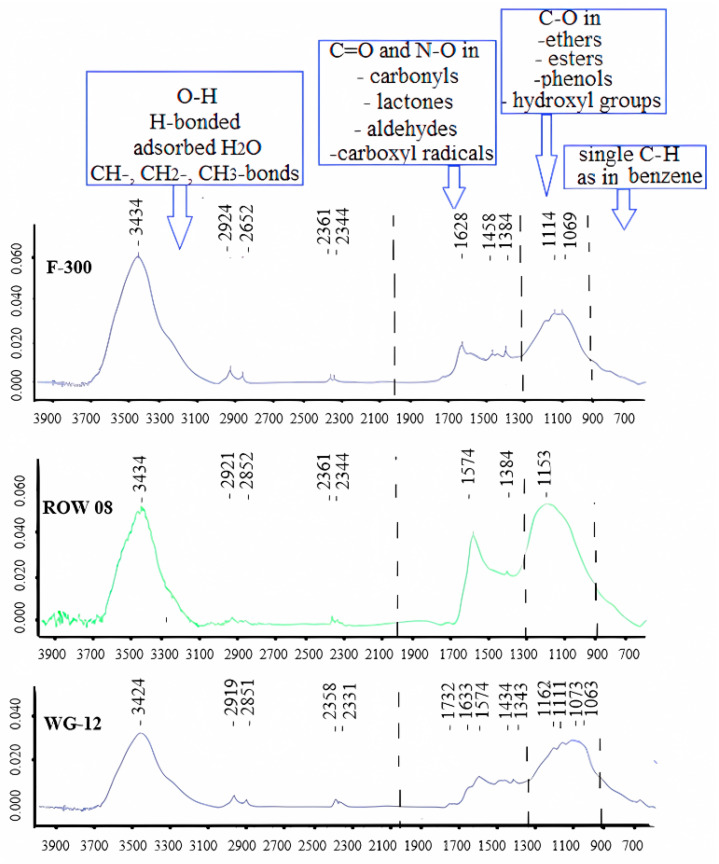
FTIR spectra for the tested activated carbons [56].

FTIR studies (Figure 9) confirm the presence of all the groups discussed above on the surface of activated carbons. Although the intensity of the band characteristics of the discussed functional groups on activated carbon WG-12 is lower than on other sorbents, it adsorbs the highest amounts of both Pb and Cd cations. However, it should be kept in mind that the FTIR study identifies the functional groups and not their amount. The amount of carboxyl and lactonic groups determined by the Bohemian method is highest on activated carbon WG-12, which also has the highest specific surface area (Table 10).

Activated carbon WG-12, which adsorbs the highest amounts of lead and cadmium cations, has the highest amounts of oxygen identified by SEM/EDX studies on its surface, while ROW 08 Supra, which adsorbs these cations the weakest, has the least amount of this oxygen (Table 12). This confirms the beneficial effect of the presence of oxygen functional groups on the adsorption volume of Pb(II) and Cd(II).

The titration method allows the number of functional groups to be determined (Boehm method). The activated carbons used in this study, produced by the steam–gas method, have both acidic and basic groupings on their surface (Table 11). Activated carbon WG-12 is the best adsorbent of lead and cadmium ions and has the largest amount of acidic oxygen groups (including carboxyl and lactone groups). ROW 08 Supra activated carbon, characterised by the smallest number of these groups, adsorbs the smallest amounts of the tested cations. The mechanism of adsorption of lead and cadmium ions with the participation of carboxyl and lactone groups discussed above is decisive.

### 2.6. Comparison of the Adsorption of the Heavy Metals Studied

Comparing the adsorption of the tested heavy metals, it can be concluded that lead is adsorbed in greater amounts than cadmium. These results are confirmed by SEM/EDS analysis (Table 10) for activated carbon WG-12. The capacities of the monolayer are as follows for sorption: Pb(II) from 135.94 to 162.19 mg/g, and for Cd(II) from 69.91 to 126.34 mg/g (Langmuir isotherm data). All the activated carbons used with very high R^2^ correlation coefficients described the adsorption of Pb(II) and Cd(II) using the Langmuir, Temkin, Toth, and Jovanovic isotherms. Lower correlation coefficients were obtained for the other isotherms tested (Freundlich, Redlich–Peterson, Halsey). Based on the analysis of R_L_ (Langmuir isotherm) and 1/n (Freundlich isotherm) values, the adsorption of all tested ions was found to be favourable. The selection of the best activated carbon depends on the ion to be removed. The most effective activated carbon for the adsorption of the Cd(II) and Pb(II) cation is sorbent WG-12.

Three models (PSO, PSO, Elovich) were used to describe the adsorption kinetics. For all cases (metal ions and activated carbons studied), the PSO model best described the kinetics process. The obtained k_2_ values (adsorption rate) were similar for Pb(II) cations (from 0.12 to 0.20 h^−1^) and Cd(II) cations (from 0.16 to 0.19 h^−1^).

An analysis of the adsorption mechanisms of Pb(II) and Cd(II) cations is presented in Section 2.5. In summary, it can be concluded that, for the adsorption of the studied ions, the adsorption process depends on the chemical structure of the surface.

The adsorption efficiency of the studied heavy metal ions from natural water is lower than from a model solution made on the basis of deionised water. The ion removal efficiency is found to depend on the concentration of competing ions. Small differences are obtained for adsorption from spring water of lead and cadmium cations (less than 3% *w*/*w*) and quite large differences from highly mineralised water (18 and 25% *w*/*w*). Adsorption of the tested heavy metals differing in charge is possible on commercial activated carbons produced by the steam–gas method. However, the adsorption efficiency depends not only on the type of activated carbon, but very much on the presence of competing ions. The greater the mineralisation of the water, the lower the adsorption efficiency.

## 3. Materials and Methods

### 3.1. Adsorbents Used in the Tests

The tests were carried out on the following three commercial carbon sorbents: Filtrasorb 300 (Chemviron, Feluy, Belgium), WG-12 (Gryfskand, Gryfino, Poland), and ROW 08 Supra (NORIT, Amersfoort, The Netherlands). These are granular carbon sorbents produced by the steam–gas method and used in water treatment plants (Table 11).

Transmission FTIR spectra (Perkin-Elmer Spectrum 2000 FTIR spectrometer, Waltham, MA, USA) were also determined. Surface topography and texture SEM-EDS studies were performed using an LEO Electron Microscopy (Carl Zeiss NTS Ltd., Oberkochen, Germany) scanning electron microscope (1430 VP). Three repetitions were performed looking at an analysed area of 500 × 400 micrometres and a surface penetration depth 5 microns. The results of these studies are presented in the work [56].

### 3.2. Solutions of the Heavy Metals Used in the Tests

To prepare lead and cadmium solutions, metal nitrates Pb(NO_3_)_2_ (chemically pure 99.999%) and Cd(NO_3_)_2_·4H_2_O (chemically pure 99.997%) by Sigma-Aldrich (Burlington, MA, USA) were used. Stock solutions of 1000 mg/L were made by dissolving the compounds in deionised water. Measurements were made using solutions at pH = 6 ± 0.2, with all the metals tested in dissolved form. At this pH, the metals take the forms of lead (Pb^2+^) and cadmium (Cd^2+^) [40]. The concentrations of Pb(II) and Cd(II) were determined using an inductively coupled plasma atomic emission spectrometer (ICP-AES Thermo, Waltham, MA, USA). Adsorption of Pb(II) and Cd(II) from natural waters was measured and compared with adsorption results of heavy metal ions from demineralised water. Four bottled waters with different mineral compositions were used, and they were enriched with Pb(II) and Cd(II) ions. Waters differing in the degree of mineralisation were used for the study, such as highly mineralised, medium mineralised, low mineralised, and spring water. The concentration of heavy metal ions tested was 10 mg/L, pH = 6. The effect of the composition of the natural waters on the adsorption of the tested ions on the WG-12 activated carbon was assessed by the adsorption efficiency.

### 3.3. Studies of Heavy Metal Adsorption Kinetics and Statics

The studies of adsorption kinetics and statics were carried out in the same way for all heavy metals. Solutions of 0.250 L were added with 0.1 g of activated carbon. The heavy metal solution with activated carbon was mixed on a laboratory shaker at 160 rpm. Tests were conducted at room temperature (T = 20 ± 1 °C). Adsorption kinetics was determined for an initial concentration of 50 mg/L for selected metals. Adsorption isotherms were determined for concentrations of 10, 20, 30, 40, 50, 60, 80, and 100 mg/L. The heavy metal concentrations used in this study allowed conditions to be obtained at which almost full saturation of the activated carbon monolayer occurred (isotherm plot parallel to the *x*-axis). Similar concentration ranges were also used by other researchers [21,37,57]. The studies of adsorption kinetics and statics were carried out using solutions at pH = 6 ± 0.2. Measurements were carried out in 4 replicates. The graphs show the standard deviation. According to many authors, this is the pH optimum during adsorption of Pb(II) and Cd(II) cations [41,58]. It was decided that the adsorption process would be carried out under these conditions, as they were close to the pH value of water treated for drinking purposes. At the same time, hydroxide precipitation of the heavy metals studied did not occur under these conditions. Adsorption kinetics was determined using samples taken after 0.5, 1, 2, 3, 4, 5, 6, 7, and 8 h. Based on the results obtained from the measurements, a six-hour time of contact between the solution and activated carbon was assumed for all heavy metals.

The effect of doses was assessed for an initial heavy metal ion concentration of 100 mg/L and activated carbon doses of 0.5, 1, 2, 3, 4, 5, and 6 g/L.

Different adsorption kinetics models belonging to the group of adsorption reaction models and the group of adsorption diffusion models were analysed. The first group analysed (adsorption reaction models) includes the pseudo-first-order, pseudo-second-order, and Elovich models [59,60]. For the adsorption diffusion models, the following were calculated: liquid film diffusion model, intraparticle diffusion model (Weber–Morris model and Bangham and Burt model) [61,62,63,64].

To describe the adsorption statics, two-parameter models (Freundlich (F), Langmuir (L), Temkin (T), Jovanovic (J), and Halsey (H)) and three-parameter models (Redlich–Peterson (R-P) and Toth (To)) were used [65]. The constants of the models were determined by non-linear regression (Excel, Solver Add-in). The degree of fit of the adsorption kinetics and isotherm models was assessed based on the correlation coefficient R^2^ and the errors [66,67]:

- Average relative error (ARE)—the Formula (5).

(5) $$\mathrm{ARE}=\frac{100}{n}\sum_{i=1}^{n}\left|\frac{q_{e,\exp}-q_{e,\mathrm{cal}}}{q_{e,\exp}}\right|$$

where:

n—number of measurement points,

q_e,exp_—adsorption capacity resulting from measurements, 

q_e,cal_—adsorption capacity calculated using a particular model.

Sum of squares of errors (SSE)—the Formula (6)

(6) $$\mathrm{SSE}=\sum_{i=1}^{n}{\left(q_{e,\mathrm{cal}}-q_{e,\exp}\right)}^{2}$$

- Chi-squared statistics (λ^2^, -)—the Formula (7)

(7) $$\lambda^{2}=\frac{100}{n}\sum_{i=1}^{n}\frac{{\left(q_{e,\exp}-q_{e,\mathrm{cal}}\right)}^{2}}{q_{e,\mathrm{cal}}}$$

- Sum of the absolute errors (SAE, -)—the Formula (8)

(8) $$\mathrm{SAE}=\sum_{i=1}^{n}\left|q_{e,\mathrm{cal}}-q_{e,\exp}\right|$$

- The hybrid fractional error function (HYBRID, -)—the Formula (9)

(9) $$\mathrm{HYBRID}=\frac{100}{n-p}\sum_{i=1}^{n}\frac{{\left(q_{e,\mathrm{cal}}-q_{e,\exp}\right)}^{2}}{q_{e,\exp}}$$

where:

p—number of isotherm parameters.

## 4. Conclusions

Commercial activated carbons used in water treatment plants for the removal of organic compounds can also be used to remove heavy metal ions. Commercial activated carbons WG-12, ROW 08 Supra, F-300, without additional modification, produced by the steam–gas method, effectively remove Pb(II) and Cd(II) cations. The adsorption capacities obtained in this study are at an average level compared to those reported in the literature.

The adsorption efficiency of Pb(II) and Cd(II) cations from natural waters is lower than that from deionised water. However, it is still high for spring and low-mineralised waters (for a metal ion concentration of 10 mg/L, the efficiency ranges from 78 to 89% *w*/*w*).

The tested heavy metals occur in a solution with pH = 6 in the form of a divalent cation. The analysis of the adsorption of Pb(II), and Cd(II) ions shows that the Pb(II) ion is best adsorbed on the activated carbons, with the highest capacity being for the WG-12 activated carbon (162.19 mg/g) and the lowest for ROW 08 Supra (135.94 mg/g). The quoted adsorption capacities were calculated using the Langmuir model, although equally high correlation coefficients were obtained for the Toth and Jovanovic isotherms, which were also used to calculate the monolayer capacities. However, the Langmuir model allows the results obtained to be compared with those quoted in the literature, as it is most commonly used, along with the Freundlich equation, to describe the adsorption process.

The analysis of different isotherm models shows that, in the case of adsorption of the studied ions, the highest correlation coefficients R^2^ and the smallest errors E were obtained for the Temkin and Langmuir models as well as for the Toth model.

Of the models analysed, the Halsey equation, describing multilayer sorption, had the lowest values of the correlation coefficient R^2^ for the activated carbons studied and for all heavy metal ions.

In this study, the adsorption kinetics was analysed and three models were used to describe it: PSO, PFO, and Elovich. The PFO and PSO models are semi-empirical but are most commonly used to describe the adsorption kinetics on activated carbons. In this study, in almost all cases, the PSO model was a better fit for the results obtained than PFO and Elovich.

As a result of the analysis of the mass transfer models, it was found that, for both lead and cadmium ion adsorption, the adsorption rate was determined by both diffusion in the liquid layer and intraparticle diffusion.

The obtained R_L_ values from the Langmuir isotherm show that the adsorption of Pb(II) and Cd(II) ions was favourable for all the activated carbons used in the study, while the sorption capacities obtained were average compared to those reported in the literature.

## Figures and Tables

**Table 1:** Constant equations of Pb(II) adsorption kinetics: pseudo-first-order, pseudo-second-order, and Elovich.

Kinetics Model	Parameter	ROW 08 Supra	WG-12	F-300
Pseudo-first-order $\frac{{\mathrm{dq}}_{t}}{\mathrm{dt}}=k_{1}\left(q_{e}-q_{t}\right)$	q_max_, mg/g	86.97	96.66	92.31
	k_1_, h^−1^	0.944	1.440	1.342
	R^2^	0.982	0.954	0.965
	ARE,	3.06	3.31	3.08
Pseudo-second-order $\frac{{\mathrm{dq}}_{t}}{\mathrm{dt}}=k_{2}{\left(q_{e}-q_{t}\right)}^{2}$	q_max,_ mg/g	99.74	106.21	102.03
	k_2_, h^−1^	0.012	0.020	0.019
	R^2^	0.995	0.997	0.997
	ARE, %	1.22	0.65	0.79
Elovich $\frac{{\mathrm{dq}}_{t}}{\mathrm{dt}}={\alpha e}^{-{\beta q}_{t}}$	α, mg/(g∙h)	293	1281	938
	Β, g/mg	0.051	0.061	0.062
	R^2^	0.959	0.946	0.943
	ARE, %	4.45	3.78	4.11

q_m_ (mg/g)—is the amount of solute adsorbed et equilibrium, qt (mg/g) is the amount of solvent adsorbed at time t, k_1_/k_2_, (1/h) is the rate constant for the pseudo-first-order/the pseudo-second-order kinetic model, α, mg/(g∙h) is the initial adsorption rate, B, g/mg reflects the number of sites available for adsorption.

**Table 2:** Constant equations of Cd(II) adsorption kinetics: pseudo-first-order, pseudo-second-order, and Elovich.

Kinetics Model	Parameter	ROW 08 Supra	WG-12	F-300
Pseudo-first-order $\frac{{\mathrm{dq}}_{t}}{\mathrm{dt}}=k_{1}\left(q_{e}-q_{t}\right)$	q_max_, mg/g	55.44	83.16	77.49
	k_1_, h^−1^	0.857	1.237	1.207
	R^2^	0.981	0.951	0.942
	ARE, %	3.66	3.93	4.42
Pseudo-second-order $\frac{{\mathrm{dq}}_{t}}{\mathrm{dt}}=k_{2}{\left(q_{e}-q_{t}\right)}^{2}$	q_max_, mg/g	64.29	92.55	86.71
	k_2_, h^−1^	0.016	0.019	0.019
	R^2^	0.996	0.995	0.995
	ARE, %	1.37	1.34	1.11
Elovich $\frac{{\mathrm{Dq}}_{t}}{\mathrm{dt}}={\alpha e}^{-{\beta q}_{t}}$	α, mg/(g∙h)	148	645	517
	Β, g/mg	0.076	0.065	0.067
	R^2^	0.969	0.946	0.969
	ARE, %	4.10	3.34	3.35

**Table 3:** Results of Pb(II) mass transfer models for the activated carbons analysed.

Models	Parameters and Correlation	WG-12	F-300	ROW 08
Weber and Morris $q_{t}=k_{\mathrm{ip}}\sqrt{t}+C$	Kp, mg · g^−1^ · h^−1/2^	19.24	19.28	23.07
	C, mg/g	51.91	47.18	31.44
	R^2^	0.8341	0.8297	0.8657
	K_1p_	43.86	44.02	46.46
	C_1_	25.15	20.14	5.39
	R^2^	0.9678	0.9769	0.9887
	K_2p_	13.63	11.62	16.31
	C_2_	68.02	67.72	50.88
	R^2^	0.9239	0.8766	0.9452
	K_3p_	1.58	3.72	0.19
	C_3_	94.72	84.27	1.31
	R^2^	0.9953	0.9032	0.9996
Bangham and Burt model log(log$(\frac{C_{i}}{C_{i}-q_{t}*m)})=\log\left(\frac{k_{b}*m}{2.303*V}\right)+\alpha *\log\left(t\right)$	K_b_	0.3698	0.3431	0.2723
	α	0.2554	0.255	0.3474
	R^2^	0.9464	0.9282	0.9463
Liquid film diffusion model $\ln\left(1-\frac{q_{t}}{q_{e}}\right)=-K_{\mathrm{LF}}*t$	K_LF_	0.8549	0.815	0.8089
	R^2^	0.977	0.9497	0.9841

kp, mg/(g∙h^0.5^)—is the intraparticle diffusion rate constant, C, mg/g—is the intercept, which is a constant related to the thickness of the boundary layer, t^0.5^—half life time, V, L—the solution work volume, m, g—is the weight of the adsorbent, k_b_ and *α*—are the model’s Bangham and Burt constants, and K_LF,_ h^−1^—parameter of liquid film diffusion model.

**Table 4:** Results of Cd(II) mass transfer models for the activated carbons analysed.

Models	Parameters and Correlation	WG-12	F-300	ROW 08
Weber and Morris $q_{t}=k_{\mathrm{ip}}\sqrt{t}+C$	K_p_, mg · g^−1^ · h^−1/2^	18.46	17.88	15.53
	C, mg/g	39.74	35.52	17.72
	R^2^	0.8624	0.8802	0.8876
	K_1p_	37.26	35.12	28.69
	C_1_	18.68	16.55	2.82
	R^2^	0.9924	0.9496	0.9915
	K_2p_	8.70	15.08	11.15
	C_2_	64.01	45.06	30.29
	R^2^	0.9948	1	0.9988
	K_3p_	1.39	1.33	1.06
	C_3_	81.49	76.31	53.32
	R^2^	0.9866	0.9581	0.9974
Bangham and Burt model log(log$(\frac{C_{i}}{C_{i}-q_{t}*m)})=\log\left(\frac{k_{b}*m}{2.303*V}\right)+\alpha_{2}*\log\left(t\right)$	K_b_	0.2999	0.2735	0.1633
	α	0.2522	0.2616	0.3416
	R^2^	0.9386	0.9397	0.9432
Liquid film diffusion model $\ln\left(1-\frac{q_{t}}{q_{e}}\right)=-K_{\mathrm{LF}}*t$	K_LF_	0.8334	0.8407	0.7935
	R^2^	0.9805	0.9737	0.9840

**Table 5:** Constants of Pb(II) adsorption isotherms on commercial activated carbons.

Isotherm Model	Activated Carbon	Constants of the Isotherm Model			Model Accuracy Parameters					
Langmuir $Q=\frac{q_{m}K_{L}C_{e}}{1+K_{L}C_{e}}$		q_m_, mg/g	K_L_, L/mg	R_L_	R^2^	ARE, %	SSE, -	λ^2^, -	HYBRID	SAE
	ROW 08	135.94	0.128	0.92–0.99	0.995	2.4	130.27	3.7	50.8	26.5
	WG-12	162.19	0.1698	0.90–0.99	0.990	6.8	38.78	0.5	7.6	13.5
	F-300	149.11	0.1548	0.91–0.99	0.995	4.1	47.84	1.2	17.6	16.4
Freundlich $Q=K_{F}C_{e}^{\frac{1}{n}}$		1/n, -	K_F_, mg/g	-	R^2^	ARE, %	SSE, -	λ^2^, -	HYBRID	SAE
	ROW 08	0.357	30.80	-	0.916	16.3	434.9	6.8	148.0	50.5
	WG-12	0.362	39.94	-	0.966	11.4	683.7	11.3	262.6	64.7
	F-300	0.356	36.08	-	0.952	12.9	503.6	8.0	175.9	55.4
Temkin $q={B\;\mathrm{lnA}}_{T}C$		A, L/mg	B, -	-	R^2^	ARE, %	SSE, -	λ^2^, -	HYBRID	SAE
	ROW 08	1.358	28.67	-	**0.998**	**0.3**	141.3	3.2	46.0	28.6
	WG-12	2.464	31.10	-	**0.998**	**0.5**	139.9	1.4	23.8	27.4
	F-300	2.008	29.53	-	**0.999**	**0.4**	96.2	1.7	26.8	25.7
Jovanovic $Q=q_{\infty }\cdot \left(1-e^{-K_{j}\cdot C_{e}}\right)$		Kj, L/mg	q_m_, mg/g	-	R^2^	ARE, %	SSE, -	λ^2^, -	HYBRID	SAE
	ROW 08	0.114	113.89	-	0.993	4.5	451.2	14.9	166.5	51.8
	WG-12	0.143	137.85	-	0.965	12.7	59.1	1.3	19.3	18.8
	F-300	0.133	126.32	-	0.975	10.1	257.9	7.6	95.3	41.7
Halsey $Q={\left(K_{h}\cdot C_{e}\right)}^{\frac{1}{n_{h}}}$		K_h,_ (mg·g^−1^/mg·L^−1^${)}^{\frac{1}{n_{h}}}$	n, -	-	R^2^	ARE, %	SSE, -	λ^2^, -	HYBRID	SAE
	ROW 08	1,922,276	3.367	-	0.894	21.3	606.6	11.4	295.4	58.1
	WG-12	1,922,276	3.182	-	0.952	17.0	868.4	16.3	460.8	71.4
	F-300	1,922,276	3.260	-	0.937	17.2	656.0	12.4	325.4	61.0
Redlich−Peterson $Q=\frac{K_{R}\cdot C_{e}}{1+a_{R}\cdot C_{e}^{\beta }}$		K_R,_ L/g	a_R_, (L/mg)^β^	Β	R^2^	ARE, %	SSE, -	λ^2^, -	HYBRID	SAE
	R0W 08	219,971	7140	0.643	0.916	16.3	434.8	6.8	177.5	50.5
	WG-12	220,024	5508	0.638	0.966	11.4	683.6	11.3	315.0	64.7
	F-300	220,010	6096	0.644	0.952	12.9	503.5	8.0	211.0	55.4
Toth $q=q_{m}\cdot \frac{b\cdot C_{e}}{{\left(1+{\left(b\cdot C_{e}\right)}^{v}\right)}^{\frac{1}{v}}}$		q_m_, mg/g	b, mg/g	v, -	R^2^	ARE, %	SSE, -	λ^2^, -	HYBRID	SAE
	ROW 08	127.60	0.115	1.216	0.997	2.1	**35.7**	**0.4**	**8.5**	**15.5**
	WG-12	209.83	0.256	0.599	0.997	2.5	**26.9**	**0.3**	**6.0**	**12.6**
	F-300	166.21	0.186	0.773	0.998	2.1	**25.5**	**0.3**	**5.5**	**12.4**

q_m_—the maximum adsorption capacity, K_L_—the constant related to the free energy of adsorption, R_L_—separator facto, 1/n—adsorption intensity, K_F_—Freundlich isotherm constant, R^2^—correlation coefficient, A—Tempkin isotherm equilibrium binding constant, B—Tempkin isotherm constant, Kj—the constant related to the free energy of adsorption, K_h_—the Halsey constant, n_h_—the Halsey constant, K_R_, a_R_, β—Redlich−Peterson isotherm constants, ν—parameter characterising the heterogeneity of the deposit, b—Toth isotherm constant.

**Table 7:** Constants of Cd(II) adsorption isotherms on commercial activated carbons.

Isotherm Model	Activated Carbon	Constants of the Isotherm Model			Model Accuracy Parameters					
Langmuir $q=\frac{q_{m}K_{L}C_{e}}{1+K_{L}C_{e}}$		q_m_, mg/g	K_L_, L/mg	R_L_	R^2^	ARE, %	SSE, -	λ^2^, -	HYBRID	SAE
	ROW 08	69.91	0.135	0.84–0.98	0.991	3.5	43.7	1.0	19.1	15.0
	WG-12	126.34	0.123	0.91–0.99	0.994	4.1	16.3	0.5	9.4	9.7
	F-300	104.42	0.131	0.89–0.99	0.992	3.8	36.2	0.7	12.1	14.8
Freundlich $q=K_{F}C_{e}^{\frac{1}{n}}$		1/n, -	K_F_, mg/g	-	R^2^	ARE, %	SSE, -	λ^2^, -	HYBRID	SAE
	ROW 08	0.282	20.10		0.871	13.3	670.3	12.06	294.2	63.1
	WG-12	0.352	28.65		0.903	17.4	222.2	5.91	130.8	36.6
	F-300	0.320	26.48		0.886	16.2	520.0	10.23	231.2	55.4
Temkin $q={B\;\mathrm{lnA}}_{T}C$		A, L/mg	B, -		R^2^	ARE, %	SSE, -	λ^2^, -	HYBRID	SAE
	ROW 08	1.585	14.05		0.996	**0.6**	145.3	1.84	32.2	27.7
	WG-12	1.214	27.23		0.997	**0.4**	88.6	2.12	38.9	23.8
	F-300	1.390	21.84		0.996	**0.5**	157.3	2.36	40.6	30.0
Jovanovic $q=q_{\infty }\cdot \left(1-e^{-K_{j}\cdot C_{e}}\right)$		Kj, L/mg	q_m_, mg/g		R^2^	ARE, %	SSE, -	λ^2^, -	HYBRID	SAE
	ROW 08	0.110	60.41		0.994	2.3	28.8	0.5	8.5	12.3
	WG-12	0.111	105.74		0.996	2.5	10.3	0.2	3.9	7.7
	F-300	0.113	88.86		0.998	2.1	10.2	0.2	3.7	8.1
Halsey $q={\left(K_{h}\cdot C_{e}\right)}^{\frac{1}{n_{h}}}$		K_h,_ (mg·g^−1^/mg·L^−1^${)}^{\frac{1}{n_{h}}}$	n, -		R^2^	ARE, %	SSE, -	λ^2^, -	HYBRID	SAE
	ROW 08	192,276	3.927		0.864	14.2	832.2	16.5	489.5	68.0
	WG-12	192,276	3.431		0.880	22.0	234.6	6.6	162.5	35.7
	F-300	192,276	3.539		0.886	9.0	574.8	12.3	322.3	56.5
Redlich−Peterson $q=\frac{K_{R}\cdot C_{e}}{1+a_{R}\cdot C_{e}^{\beta }}$		K_R,_ L/g	a_R_, (L/mg)^β^	Β	R^2^	ARE, %	SSE, -	λ^2^, -	HYBRID	SAE
	ROW 08	219,875	10941	0.717	0.871	13.3	670.3	12.1	352.9	63.1
	WG-12	220,038	7678	0.648	0.903	17.4	222.0	5.9	157.0	36.6
	F-300	220,016	8307	0.680	0.886	16.2	519.9	10.2	277.3	55.4
Toth $q=q_{m}\cdot \frac{b\cdot C_{e}}{{\left(1+{\left(b\cdot C_{e}\right)}^{v}\right)}^{\frac{1}{v}}}$		q_m_, mg/g	b, mg/g	v, -	R^2^	ARE, %	SSE, -	λ^2^, -	HYBRID	SAE
	ROW 08	65.97	1.307	0.109	**0.998**	1.7	**12.0**	**0.3**	**5.0**	**9.0**
	WG-12	114.46	0.104	1.397	**0.998**	2.2	**4.0**	**0.1**	**2.1**	**5.1**
	F-300	94.33	0.104	1.500	**0.999**	1.2	**3.7**	**0.1**	**1.6**	**4.5**

**Table 9:** Removal efficiency of Pb(II) and Cd(II) ions from natural waters on WG-12 activated carbon, C_0_ = 10 mg/L.

Water	Water Composition, mg/L								TM	Adsorption Efficiency, % *w*/*w*	
	Anions				Cations						
	${\mathrm{HCO}}_{3}^{-}$	${\mathrm{SO}}_{4}^{2-}$	F^-^	Cl^-^	Ca^2+^	Mg^2+^	Na^+^	K^+^		Pb(II)	Cd(II)
A	-	-	-	-	-	-	-	-	-	92.9 ± 2.0	81.8 ± 1.7
B	168.00	14.71	0.09	2.80	50.10	6.08	2.50	1.19	258.27	90.8 ± 2.4	79.1 ± 1.6
C	186.70	43.62	-	3.19	44.09	17.01	11.10	1.00	322.21	89.2 ± 2.8	78.2 ± 2.1
D	432.7	-	0.23	2.50	102.2	16.00	11.25	2.34	592.32	83.7 ± 3.1	70.8 ± 1.6
E	1403.7	32.0	-	7.0	180.9	142.7	63.0	7.5	1836.80	67.8 ± 1.7	63.7 ± 2.4

TM—total mineralisation, A—demineralised water, B—spring water, C—low-mineralised water, D—medium mineralised water, E—highly mineralised water.

**Table 10:** SEM/EDS analysis results (mass percent, %).

Spectrum	C	O	Na	Mg	Al	Si	S	K	Ca	Fe	Cr	Pb	Cd
WG-12 + Pb(II)	60.96	25.35	-	-	1.41	1.53	0.24	1.70	1.32	0.97	-	6.53	-
	57.64	28.78	-	-	1.87	1.94	0.24	1.88	0.54	1.45	-	5.64	-
	46.05	36.66	-	-	1.57	3.15	-	0.76	0.54	3.49	-	7.78	-
	55.10	31.02	-	-	1.07	2.17	-	0.59	0.76	0.96	-	8.33	-
WG-12 + Cd(II)	71.05	24.63	-	-	1.07	1.00	-	-	-	0.65	-	-	0.50
	62.46	30.39	-	-	1.67	2.28	-	-	-	0.73	-	-	0.73
	70.79	24.79	-	-	1.02	0.99	-	-	-	0.57	-	-	0.76
	60.40	32.88	-	-	1.38	1.62	0.82	-	-	1.09	-	-	0.61

**Table 11:** Physical and adsorption properties of activated carbons used in the research [53,54,55].

Activated Carbon	Unit	WG-12	ROW 08 Supra	F-300
Bulk density, (PN-EN 12915)	g/dm^3^	424 ± 27	381 ± 16	542 ± 36
pH of the water extract (PN-82/C-97555)	-	6.7	8.6	6.8
Specific surface area	m^2^/g	1098 ± 38	897 ± 30	847 ± 29
Pore structure				
V_total,_	cm^3^/g	0.990	1.135	0.987
V_macr._	cm^3^/g	0.400	0.246	0.217
V_mezo._	cm^3^/g	0.066	0.453	0.294
V_micr._	cm^3^/g	0.524	0.436	0.476
Iodine adsorption. LI, (PN-EN 12902)	mg/g	1050	1096	1065
Grain composition—sieve analysis	%			
(PN-EN 12902)				
> 2.0 mm		4.9	36.4	31.4
2.0 ÷ 1.5 mm		57.4	41.2	23.4
1.5 ÷ 1.0 mm		34.3	21.7	30.2
1.0 ÷ 0.5 mm		2.2	0.3	10.2
<0.5 mm		1.0	0.1	4.6
pH_PZC_		6.4	6.5	6.6
Acidic groups (Boehm method)	mmol/g	0.586	0.434	0.544
- carboxylic group		0.182	0.063	0.138
- lactonic group		0.209	0.120	0.048
- phenolic groups		0.110	0.409	0.316
- carbonyl groups		0.085	0.021	0.060
Basic groups/sites		0.467	0.592	0.512

**Table 12:** SEM/EDS analysis results (mass percent, %).

Spectrum	C	O	Na	Mg	Al	Si	S	K	Ca	Fe
F-300	66.32	29.92	-	-	1.18	1.14	0.85	-	-	0.59
	63.72	31.70	-	-	1.53	1.51	0.73	-	-	0.80
	66.15	29.98	-	-	1.24	1.24	0.83	-	-	0.56
ROW 08 Supra	70.10	26.00	-	-	0.57	0.98	0.80	-	0.87	0.74
	72.6	24.66	-	-	0.34	0.44	0.75	-	0.56	0.55
	72.60	25.15	-	-	0.33	0.92	0.75	-	0.52	0.52
WG-12	51.13	38.88	-	-	0.90	6.70	0.20	-	-	1.08
	48.63	40.88	-	-	2.88	4.18	0.20	-	-	1.78
	45.75	42.56	-	-	3.40	4.89	0.27	-	-	1.83

## Data Availability

Data are contained within the article.
